# Research in Epidemiology in Brazil: challenges, pathways, and commitments for the future

**DOI:** 10.1590/1980-549720250003.supl.1

**Published:** 2025-11-17

**Authors:** Antonio Fernando Boing, Maria de Jesus Mendes da Fonseca, Margareth Guimarães Lima, Claudia Leite de Moraes, Katia Vergetti Bloch

**Affiliations:** I Universidade Federal de Santa Catarina, Graduate Program in Public Health – Florianópolis (SC), Brazil.; II Fundação Oswaldo Cruz, National School of Public Health, Department of Epidemiology and Quantitative Methods – Rio de Janeiro (RJ), Brazil.; III Universidade Estadual de Campinas, School of Medical Sciences, Department of Public Health – Campinas (SP), Brazil.; IV Universidade do Estado do Rio de Janeiro, Hesio Cordeiro Social Medicine Institute, Department of Epidemiology – Rio de Janeiro (RJ), Brazil.; V Universidade Federal do Rio de Janeiro, Institute of Public Health Studies – Rio de Janeiro (RJ), Brazil.

**Keywords:** Epidemiology, Health research agenda, Brazil, Public health

## Abstract

In this article, we discuss the challenges and perspectives of Brazilian Epidemiology based on the reflections presented in the Fifth Strategic Plan for the Development of Epidemiology in Brazil (2025–2029). The importance of a scientific agenda committed to quality, innovation, equity, and social transformation is highlighted. We also ponder that Epidemiology must reaffirm its identity as a critical, collaborative, and socially-referenced field. Brazilian Epidemiology must carry on its mission of contributing to the response to health crises, to the reduction of health inequities, and supporting the construction of a national project of sustainable development. Together with the analysis of the significant advances achieved throughout its history, the challenges of research in Epidemiology are debated, such as the chronic underfunding of research in the country, the regional and thematic inequalities in the distribution of resources, academic productivism, the need to bring science closer to the population, and to expand the theoretical and methodological density of studies. Simultaneously, we highlight that opportunities are emerging related to the expansion of open science, the democratization of access to data, and the expansion of the social relevance of research.

## PRESENTATION

Research in Epidemiology has played a key role in understanding health dynamics in Brazil, allowing not only to describe historical trajectories of morbidity and mortality indicators, but also to analyze regional and social variations in patterns of illness and use of services. The accumulation of knowledge that has been brought by it has been fundamental to consolidate evidence about the Brazilian epidemiological transition, marked by the persistence of infectious diseases, the expansion of chronic diseases, and the overlap of diseases resulting from overall violence and working conditions.

This movement has enabled the advancement of scientific knowledge and the formulation of effective and equitable public policies, in addition to supporting actions of social control. By articulating theory, method, and practice, Brazilian Epidemiology is constituted not only as a technical discipline, but also as a strategic instrument to contribute to the development of a fairer society. Its value has lain in the ability to combine the well-known theoretical and methodological rigor with social commitment, guiding interventions that seek equity, quality of life, and universality in access to health.

The centrality and importance of epidemiological research have been strengthened in the 21st century. National scientific production has grown in volume and diversity in recent decades, consolidating large population-based surveys and the wide use of official record systems. More recently, it was decisive in the production of knowledge of COVID-19, when it demonstrated the capacity for rapid mobilization, coping with disinformation campaigns, and providing support to society in demanding effective public policies. However, these advances coexist with challenges that permeate science on a global and national scale: the diffusion of anti-science discourses, the instability of resources aimed at research^1^, and regional inequalities in the distribution of funding and installed capacity^2^. The debate on the continuous need for our improvement as researchers — from those already consolidated to the new generations — is also relevant, concerning theoretical and methodological advances in the research carried out and the limits of academic productivism with its distance from social needs.

Simultaneously, opportunities are open for strengthening the field. The advancement of research aligned with the needs of the population, with critical and qualified training of researchers, the adoption of open science practices^3^, the establishment of inter-institutional networks in the country and international partnerships, including the South-South Cooperation, the approximation between researchers and the surveyed populations, and the critical and productive use of technological innovations provide ways to expand the reach and social relevance of epidemiological research. The incorporation of these elements, anchored in solid theoretical-methodological references and guided by ethical principles and commitment to the population, can enhance the ability of Epidemiology to produce transformative knowledge. The history of Brazilian Epidemiology is very rich and consistent, which increases everyone's responsibility to carry on the advances and build a future in which science remains critical, socially-referenced, and capable of facing the health and political crises that challenge the country.

Reflecting on the challenges and opportunities in research presented to epidemiologists is key to our practice to enable planned actions and with concrete and collectively-established goals. Therefore, the Fifth Strategic Plan for the Development of Epidemiology in Brazil (2025–2029), elaborated based on consultations with the community of epidemiologists in the academic institutions, health management and services of the Brazilian Unified Health System (SUS), in several debates, with dozens of participants and over two years, dedicated a thematic area to research in Epidemiology. In it, three central axes guide the diagnosis of the challenges to be faced and the actions to be developed to overcome them:

- social inequalities and inequities in health;
- funding for research and research institutions;
- theory, method, and ethics in research.

In this article, which accompanies the publication of the Fifth Strategic Plan for the Development of Epidemiology in Brazil (2025–2029), we contextualize and discuss some of the aspects presented in the document.

### From instability to the consolidation of a robust science project in Brazil

Scientific research is one of the pillars for the economic, social, and technological development of any nation. In Brazil, however, funding in Science and Technology (S&T) faces an instability scenario that has been marked by occasional advances and frequent setbacks. These movements compromise the continuity of projects, the consolidation of research groups, and the maintenance of researchers in the country. Understanding this context requires a critical historical, political, and institutional perspective, and reversing it requires a strong articulation of different sectors of society, including epidemiologists.

Brazil has developed an important institutional framework in the S&T field over decades. Between the years 2000 and 2013, there was a cycle of funding expansion with the creation of sectoral funds and the consolidation of the National Development, Scientific and Technological Funding (*Fundo Nacional de Desenvolvimento Científico e Tecnológico* – FNDCT), expanding the resources available to the sector. But, subsequently, there was a significant decrease in investments. Between 2013 and 2020, federal resources in S&T fell 37% in real terms^1^. In 2020, the budgets of the National Council for Scientific and Technological Development (*Conselho Nacional de Desenvolvimento Científico e Tecnológico* – CNPq) and FNDCT, added together, became smaller than those recorded in the early 2000s. In fact, between 2000 and 2020, the corrected budget of the Brazilian Ministry of Science, Technology and Innovation was reduced by 52% and the Ministry of Education, by 50%^1^. Conversely, the expenditure of the Coordination for the Improvement of Higher Education Personnel (*Coordenação de Aperfeiçoamento de Pessoal de Nível Superior* – Capes) dropped back to levels close to 2011. Despite recomposition efforts after this period, such as the approval of Complementary Law No. 177/2021, which sought to ensure the full implementation of the FNDCT resources, and improvements in budgetary documents in 2023 and 2024 compared to previous years, structural imbalances, instability, and underfunding still prevail. In order to illustrate that, between 2021 and 2024, the expenses paid by Capes grew in absolute terms only at BRL 1.2 billion^4^ and by CNPq, at BRL 0.5 billion^5^.

This national context of setbacks with palliative advances results in the weakening of the training of researchers and the national scientific basis, while deepening inequalities in scientific production and capacity for innovation among the different regions of the country, increasing external dependence and undermining the conditions of Brazil to transform science into sovereign development. We can mention brain drain and the absence of structuring policies for settling young doctors as examples. More than recomposing budget values or having specific programs, it is necessary to rethink the place of science in the public agenda, breaking with the logic of funding as a variable of fiscal adjustment and affirming it as strategic investment.

Given this scenario, the Fifth Strategic Plan for the Development of Epidemiology in Brazil (2025–2029) defines as one of its priorities the struggle for the formulation of more robust and stable funding policies for epidemiological research and that expand research whose authors investigate the impacts of public investment in S&T on the development of the country. Not only marginal additions to the budget allocation of agencies and ministries or discourses to enhance scientific research are necessary. An articulated set of actions, both immediate and long-term, capable of shielding science from political instabilities and consolidating it as a structuring axis of national development, ensuring it is a real priority on the public agenda, is imperative.

In addition to enabling robust funding, ensuring equitable access to it is essential to promote diversity, innovation, and scientific excellence. Despite advances, inequalities persist both in terms of topics and beneficiaries. These disparities hamper advances in some neglected topics and also individual careers, limiting the diversity of essential perspectives to scientific discoveries^6^.

A clear example are studies on neglected tropical diseases in Brazil. At the same time research funding for neglected tropical diseases has stagnated over the years in the country, with insufficient funding for diseases with high prevalence, load, and mortality rate^7^, there is a lack of correlation between disease burden, scientific production, and government funding^8^. For instance, among the studies published between 2012 and 2016 on the subject, there is emphasis on basic biomedical research and a lack of studies on policies, health systems, and Social Sciences.

Between 2007 and 2016, government funding for cancer research in Brazil, which amounted to almost USD 489 million destined for 7,622 projects and 3,068 researchers, showed a strong regional concentration: the Southeast received 84.4% of these resources^9^. More recently, according to FNCDT data, 93% of its refundable resources estimated for 2023 of previously-contracted projects were concentrated in the Southeast and South regions^10^. Among the nonrefundable resources of 2022 and 2023, it was verified that from 69 to 75% of the contracted values of projects that ended in the period were concentrated in the South and Southeast, compared to only 2 to 6% in the North^11^. The FNCDT distribution of scholarships in 2023 concentrated in the South and Southeast 66% of junior postdoctoral fellowships, 72% of scientific initiation, and almost 60% of industrial technological development^11^.

In short, the Fifth Strategic Plan for the Development of Epidemiology in Brazil (2025–2029) calls for Brazil to take on a bolder position in S&T funding, with a structuring and ambitious project. It is proposed that we should be able to leave as a legacy for the following years a transformative and solid S&T proposal, with broad social support and less vulnerable to cycles of austerity and dismantling attempts.

### Agendas, methods and meanings of research in Epidemiology in Brazil

In addition to funding, we should widely discuss the issues we have investigated and how we have developed these studies in our field. We can consider scientific congresses as an introduction, as they play a relevant role in defining and evidencing research agendas. In the case of Epidemiology in Brazil, the congresses of the Brazilian Association of Collective Health (*Associação Brasileira de Saúde Coletiva* – Abrasco) in the field organize the presentation of works on thematic axes, which work as a mirror of the priorities and challenges of the area. In the comparison between the Brazilian Epidemiology congresses of 2017 and 2024, we verified great stability of these axes, such as theory and methods in Epidemiology; evaluation of policies and services; education and training; health throughout the course of life; chronic diseases; violence and accidents; and studies on specific populations (Indigenous, Black, LGBTQIAPN+ people, those deprived of liberty, migrants, and refugees). More recently, there has been the inclusion or expansion of topics such as climate crisis and health, public health emergencies, aging, and work-related injuries. Despite these advances, significant gaps persist in the face of the emerging and latent needs of society.

The consolidation of major national research, such as the National Survey of Health^12^, the National Survey of School Health^13^, the Consumer Expenditure Survey^14^, and the Surveillance System of Risk and Protective Factors for Chronic Diseases by Telephone Survey^15^ have leveraged, with great relevance and need, research on chronic noncommunicable diseases (NCDs) and their care and risk factors, especially considering the two Plans of Action for Tackling NCDs, of the Ministry of Health, one with 2011 to 2022 goals^16^ and the other, with 2021 to 2030 goals^17^.

Furthermore, we have some ongoing longitudinal studies that constitute benchmarks for understanding determinants of health and disease. As examples of several other studies of considerable relevance, we mention the Pelotas Birth Cohort, which started in 1982 and has new periodic waves since then, that accompanies individuals from birth enabling analyses on child development, adolescence, and adulthood^18^; the Longitudinal Study of Adult Health (*Estudo Longitudinal de Saúde do Adulto* – ELSA-Brasil), which started in 2008, aimed at the adult population, with emphasis on NCDs, particularly cardiovascular and metabolic diseases^19^; and the Brazilian Longitudinal Study of Aging (*Estudo Longitudinal da Saúde dos Idosos Brasileiros* – ELSI-Brasil), which investigates health, functional, and social conditions of older adults^20^. Investigating official databases in Brazil, there is also the Center for Data and Knowledge Integration for Health (*Centro de Integração de Dados e Conhecimentos para Saúde*)^21^.

Conversely, mental health-related and violence-related injuries — priority issues in the discussions of the Fifth Strategic Plan for the Development of Epidemiology in Brazil (2025–2029) for its high and growing burden on the Brazilian population and the hundreds of thousands of leaves of absences from work — still have fewer studies in view of its epidemiological relevance. For instance, in 2015, these disorders accounted for 9.5% of the total disability-adjusted life years (DALY), occupying the third and first position in the DALY classification, and years of life lost due to premature mortality, respectively, with emphasis on depressive and anxiety disorders^22^. According to a report by the Ministry of Public Labor Prosecution and the Office of the International Labor Organization for Brazil, the benefits due to temporary disability associated with mental health at work more than doubled between 2022 and 2024, from almost 201 thousand to more than 471 thousand (an increase of 134%)^23^.

The estimates of violence are also frightening. According to data from the Notifiable Diseases Information System, only in 2023 there were 588,388 cases of interpersonal/self-inflicted violence in Brazil^24^. In turn, according to the Public Security Map of 2025, although Brazil recorded, in 2024, a 6.3% reduction in intentional homicides compared to the previous year (35,365 victims, accounting for 37,754 in 2023), the magnitude remains unacceptable, and violence against women is increasing and manifests in different forms. In 2024, for example, there were records of 2,422 homicides of women, 1,459 femicides, and 71,834 cases of rape of women, which would correspond to an average of 196 cases per day.

For years, the maintenance of the high burden of infectious diseases in Brazil and, more recently, the COVID-19 pandemic, have also reinforced the importance of surveillance studies and those on communicable diseases^25^, which should be constantly monitored and require such action. While neglected diseases remain challenging^26^, research topics — such as climate change^27^ and public health emergencies^28^ — should also be extensively researched. It is worth developing studies on topics such as response to disasters — outbreaks and epidemics, in dialogue with surveillance and including society —, description and evaluation of health policies of people deprived of liberty, health of formal and informal workers, health of Indigenous, quilombola, and traditional populations, emerging and reemerging communicable diseases, mental health, violence, and other external causes. It is challenging, though necessary, to enable this expansion, while strengthening research on chronic diseases, which correspond to the highest proportion of deaths in the country.

Another aspect worth highlighting is the continuous need for advances in theoretical models capable of sustaining and guiding the interpretation of epidemiological data. It is imperative that research does not rely on mechanical applications of statistical models and with little conceptual reflection, which prevents the contextualization of the findings and the expansion of their social relevance^29-32^. In terms of social inequalities in health, particularly, it is noteworthy that scientific production cannot be limited to the mere test of statistical significance in the associations between socioeconomic variables and health outcomes. Authors of a previous study showed that, despite its increasing presence in the literature, the field of health inequalities still presents a shortage of longitudinal, multidimensional, and interdisciplinary approaches, making it difficult to understand the mechanisms that produce and reproduce inequalities^30^.

Likewise, statistical tests, an important part of epidemiological analyses, should be subordinated to a theoretical model, to an understanding of the economic, social, and cultural issues that shape the relations studied. Understanding the causes and obstacles that sustain social inequalities in health requires more than the important empirical description of disparities; seeking to understand their driving forces and consequences is necessary. The theory, in this context, is not accessory, but essential to guide analyses and subsidize actions aimed at equity^33^. Particularly, it is necessary to critically understand the effects on population health of income concentration processes and the implementation of neoliberal and austerity policies, in addition to those that are unjust and inefficient. This endeavor involves the solid training in data theory and analysis in graduate programs — with critical researchers working in a transdisciplinary and creative way —, the consolidation of research agendas that propose conceptual and methodological advances, and the strengthening of research with critical, innovative, robust, and theoretically-grounded analyses.

In turn, research on race inequalities in health or racism and health is still under development, with an increase in the last decade^30,32^. According to researchers^32^, the fields with more publications on the health of the Black population were Public Health, with 21.6% of the total studies analyzed, Nursing, Collective Health, Social Sciences and Applied Social Sciences, and Psychology. Epidemiology accounted for 6% of the total research in the analyzed period. Studies whose authors address social inequalities in health often include race/skin color in their analyses, but those that focus on the effect of race/skin color on health are rare. Therefore, it is worth stating that although research has substantially advanced in this topic, investments in studies in the field and a critical view of racism in society are still needed.

It is also worth mentioning the scarcity of investigations with specific groups or those in vulnerability, such as the Indigenous^34^, quilombola, riparian^35^, and LGBTQIAPN+ populations. Although two thematic supplements from 2024 — one from the *Revista Brasileira de Epidemiologia* [Brazilian Journal of Epidemiology]^36^ and one from the *Epidemiologia e Serviços de Saúde* [Epidemiology and Health Services] journal^37^ — were exclusively intended for the health of the transgender population, it is important to expand scientific production with vulnerable groups. Overall, we should be committed to think of research not on some population groups, but with and for them.

While one aspect of these challenges involves research funding and the collective creation of the national priority's agenda, another aspect refers to encouraging an academic productivism that often presents itself disconnected from the needs of society. Criticism has long been accumulated about the limitations of bibliometric indicators that evaluate the "impact" of scientific production by citations only^38,39^. The deleterious effects that evaluation criteria essentially based on the number of publications may have on the autonomy, quality, research agenda of researchers, and their health are also questioned^40^. Another aspect that has been worrisome for some time is the proliferation of research whose authors do not seek originality^41^, diversification or creativity, because the academic environment and its rewards are more strongly tied to the quantity published and less to quality, relevance, or innovation. Although we can be prolific and dynamic in scientific production, as it is our mission to produce and disseminate knowledge, our research must be guided by the needs of the population and the commitment to effectively contribute to the advancement of knowledge. To this end, we need collaborative environments, interinstitutional research networks, and robust funding. In this regard, we need to unite our efforts to formulate policies of evaluation and scientific recognition based on the concrete challenges of our society. This involves rethinking the assessment of graduate programs, the selection processes for professors and researchers, the lines of development and project selection criteria as well as the journals in which we publish our findings. At least since the 2017–2020 quadrennium, in the field of Collective Health, the evaluation criteria of Capes have been prioritizing the quality of training and the social impact of graduate programs. More recently, the qualitative evaluation of the faculty's scientific publications was also included. Such changes have the potential to induce improvements in intellectual production and in the training of future researchers. We must continue advancing and collectively taking the next steps in order to seek the alignment of our goals with our praxis.

### Knowledge in motion: open science, translation, and democratization in Brazilian Epidemiology

Brazil occupies a prominent position in scientific production in Health and Epidemiology, as aforementioned, however a contradiction persists: part of this knowledge is not disseminated outside the academic environment and in many cases does not translate into agile, effective, and equitable political decisions. As previously stated, part of the challenge lies in establishing an agenda according to social needs. Nevertheless, another aspect involves the need to present to managers, health professionals, social movements, and society in general the production of science as something understandable and applicable. Although Brazilian epidemiologists produce high-quality, socially-committed, and admirable research — even in the face of enormous structural challenges —, it is essential to create effective mechanisms for translation and dissemination of knowledge that can make it reach people and have meaning for different actors. Democratizing Epidemiology, therefore, is not restricted to opening data or publishing articles, but requires rethinking the speed of scientific production, communication formats, creating reliable and accessible repositories, and investing in methods for popularizing science.

In some cases, the time of science does not follow the urgency of the formulation of public policies, which can contribute to decisions based on impressions or incorrect information, not evidence. Although many research requires a long period of development, it is paramount that the scientific community is able to offer rapid responses that meet pressing needs, whether in public health emergencies — such as outbreaks, pandemics, natural disasters — or any other need of social interest.

Overcoming these barriers requires understanding the translation of knowledge as a structuring practice of Epidemiology, which means developing languages, products, and methods capable of making epidemiological evidence actionable by different audiences. In addition to scientific articles, we highlight executive reports and interactive panels for managers, podcasts, videos and infographics for society, materials adapted for schools and community collectives that bring research closer to its recipients, recognizing them as coproducers of meaning and solutions. In this sense, the Fifth Strategic Plan for the Development of Epidemiology in Brazil (2025–2029) emphasizes the need to train professionals in scientific popularization techniques, increasing the visibility, transparency, and circulation of knowledge beyond the academic walls.

At the same time, we must think about the ways in which scientific articles are disseminated. It will be important to expand discussions and actions toward open science, involving political and technical aspects related to the use of reliable repositories of epidemiological data, protected by the Brazilian General Data Protection Law (*Lei Geral de Proteção de Dados Pessoais* – LGPD). Practices to increase the transparency and reproducibility of science further strengthen and legitimize it. Therefore, we should aim at the expansion of open-access publication, the sharing of data and database analysis routines, and transparency in review processes. Brazil, especially by the Scientific Electronic Library Online (SciELO) initiative, has a strong history of publications with open access. Collective Health journals published in Brazil and that disclose the production of Brazilian Epidemiology also undertake the mission of widely disseminating knowledge; however, open access does not always mean equity or justice in scientific dissemination. A significant volume of Brazilian public resources has been directed to the payment of publication fees in foreign journals, reorienting investments in Brazilian science to large business conglomerates. At the same time, CNPq funding calls supporting editorial work provide each selected Brazilian journal with an amount equivalent to the cost of two processing fees in foreign journals^42^. Rethinking the dissemination of knowledge produced in Brazil, reconciling the important internationalization of our production, its translation and application in our society, the strengthening of journals edited in the country, and the fair and rational use of public resources and the approximation of society is the mission of Brazilian science, in which Epidemiology must have a central role.

If broad access to publications is a fundamental step toward democratizing scientific knowledge, it is equally essential to ensure open, democratic, and transparent access to secondary data of epidemiological interest throughout its scope. This practice strengthens social control, expands the legitimacy of policies, and creates conditions for a rich plurality of analyses by different social actors. Especially in research funded with public resources and in the databases of official records, it is necessary that public agencies make efforts to enable broad access to data — whether from an information system, or from specific research, or derived from the linkage of databases. It is important that access to them is open to all researchers and research groups with legitimate interest. To this end, the debate with the scientific community and social control should be continuous, and data security, as provided for in the LGPD, should be guaranteed. Likewise, the Fifth Strategic Plan for the Development of Epidemiology in Brazil (2025–2029) reinforces that research projects funded by the Ministry of Health and other public agencies are primarily selected through widely-publicized public notices, while direct contracting of research groups follows clear and public criteria restricted to specific and strategic situations.

There is a unique opportunity in the country with the consolidation of the National Health Data Network (*Rede Nacional de Dados em Saúde*), with important initiatives in digital health, wide use of electronic records at different levels of health care, extensive home research and health services, added to the development of analytical techniques for large databases, artificial intelligence, and machine learning. Associating these potentialities with the deepening of democratization in safe and responsible access to data and research questions relevant to Brazilian society is a healthy challenge that Epidemiology should take on.

Likewise, it is necessary to face the asymmetry of possibilities between regions and municipalities in the analysis of data. While some have teams trained to interpret complex data, others face technical difficulties for the strategic use of available information. In this gap, misguided policies and misinformation find fertile ground, reducing the potential for impact of epidemiological production. Therefore, it is worth supporting the ongoing learning of surveillance teams and other health professionals for data collection and entry in information systems, encouraging the consolidation of strategic information centers in surveillance and stimulating the integration between municipalities, health regions, and states. Simultaneously, there should be support to young researchers and professionals with insertion in public health services to develop and submit competitive proposals in development notices.

Democratizing epidemiological knowledge is a political commitment to Brazilian society, to the social policies of the country, and to reducing inequalities. It means ensuring that science guides policies more quickly and effectively, but also that it is appropriate for those living in vulnerable territories and historically-invisible collectives. Brazilian Epidemiology is able to lead this transformation, and it is important that it incorporates the translation of knowledge and open science as axes of its practice.

## CONCLUSION

Considering these reflections, research in Epidemiology should be understood as an expression of a social commitment that goes through the scientific domain and beyond. It must be a way to propose an integration between the academia, health services, managers, and society, so that a public, critical, and transformative science can be consolidated.

Research in Epidemiology in Brazil is faced with the challenge of overcoming the structural limitations imposed by underfunding and historical inequalities, while increasing its capacity to produce critical, quality, and innovative knowledge, socially-referenced and oriented toward transformation. The Fifth Strategic Plan for the Development of Epidemiology in Brazil (2025–2029) points out some paths that seek to strengthen Epidemiology and invites the entire scientific community and society, overcoming institutional and disciplinary boundaries, to build a collaborative and supportive science that gathers innovation, technical rigor, and social commitment.

## Data Availability

does not apply.
