# ERRATUM: Research in Epidemiology in Brazil: challenges, pathways, and commitments for the future

**DOI:** 10.1590/1980-549720250003.supl.1erratum

**Published:** 2026-03-09

In the manuscript "Research in Epidemiology in Brazil: challenges, pathways, and commitments for the future", DOI: https://doi.org/10.1590/1980-549720250003.supl.1, published in the Rev. bras. Epidemiol. 28 (suppl 1) • 2025.

**Where it reads:**

Antonio Fernando Boing, Maria de Jesus Mendes da Fonseca, Margareth Guimarães Lima, Claudia Leite de Moraes, Katia Vergetti Bloch

**It should read:**

Antonio Fernando Boing, Maria de Jesus Mendes da Fonseca, Margareth Guimarães Lima, Katia Vergetti Bloch, Claudia Leite de Moraes

Scientific Editor: Maria Fernanda Tourinho Peres https://orcid.org/0000-0002-7049-905X

