# Supplementary material for: Research in Epidemiology in Brazil: challenges, pathways, and commitments for the future
Source: Rev Bras Epidemiol. 2025 Nov 17;28(Suppl 1):e250003supl1. doi: 10.1590/1980-549720250003.supl.1 (PMC12622984; doi:10.1590/1980-549720250003.supl.1)
Supplement: Translation

# Translation: Pesquisa em epidemiologia no Brasil: desafios, caminhos e compromissos para o futuro

**DOI:** 10.1590/1980-549720250003.supl.1.2

**Authors:** Antonio Fernando Boing, Maria de Jesus Mendes da Fonseca, Margareth Guimarães Lima, Claudia Leite de Moraes, Katia Vergetti Bloch

**Affiliations:** I Universidade Federal de Santa Catarina, Programa de Pós-Graduação em Saúde Coletiva – Florianópolis (SC), Brasil.; II Fundação Oswaldo Cruz, Escola Nacional de Saúde Pública, Departamento de Epidemiologia e Métodos Quantitativos – Rio de Janeiro (RJ), Brasil.; III Universidade Estadual de Campinas, Faculdade de Ciências Médicas, Departamento de Saúde Coletiva – Campinas (SP), Brasil.; IV Universidade do Estado do Rio de Janeiro, Instituto de Medicina Social Hesio Cordeiro, Departamento de Epidemiologia – Rio de Janeiro (RJ), Brasil.; V Universidade Federal do Rio de Janeiro, Instituto de Estudos de Saúde Coletiva – Rio de Janeiro (RJ), Brasil.

**Keywords:** Epidemiologia, Agenda de pesquisa em saúde, Brasil, Saúde pública

## Abstract

Este artigo discute os desafios e as perspectivas da epidemiologia brasileira com base nas reflexões apresentadas no V Plano Diretor para o Desenvolvimento da Epidemiologia no Brasil (2025–2029). Destaca-se a importância de uma agenda científica comprometida com a qualidade, a inovação, a equidade e a transformação social. Também se sustenta que a epidemiologia deve reafirmar sua identidade como campo crítico, colaborativo e socialmente referenciado. A epidemiologia brasileira deve continuar com sua missão de contribuir com o enfrentamento às crises sanitárias, colaborar para a redução das iniquidades em saúde e apoiar a construção de um projeto nacional de desenvolvimento sustentável. Com a análise dos avanços expressivos conquistados ao longo de sua história, debatem-se desafios da pesquisa em epidemiologia, como o subfinanciamento crônico da pesquisa no país, as desigualdades regionais e temáticas na distribuição de recursos, o produtivismo acadêmico, a necessidade de aproximar a ciência da população e de se ampliar a densidade teórico-metodológica dos estudos. Ao mesmo tempo, destaca-se que emergem oportunidades ligadas à ampliação da ciência aberta, à democratização do acesso a dados e à ampliação da relevância social das pesquisas.

## APRESENTAÇÃO

A pesquisa em epidemiologia tem desempenhado papel central na compreensão das dinâmicas de saúde no Brasil, permitindo não apenas a descrição de trajetórias históricas de indicadores de morbimortalidade, mas também a análise de variações regionais e sociais nos padrões de adoecimento e de uso de serviços. O acúmulo de conhecimento que ela tem trazido tem sido fundamental para consolidar evidências sobre a transição epidemiológica brasileira, marcada pela persistência de doenças infecciosas, pela expansão das doenças crônicas e pela sobreposição de agravos decorrentes das violências em geral e das condições de trabalho.

Esse movimento tem permitido avançar no conhecimento científico e na formulação de políticas públicas efetivas e equânimes, além de apoiar ações do controle social. Ao articular teoria, método e prática, a epidemiologia brasileira constitui-se não somente como disciplina técnica, mas também como instrumento estratégico para contribuir com a construção de uma sociedade mais justa. Seu valor tem residido na capacidade de combinar o conhecido rigor teórico-metodológico com compromisso social, orientando intervenções que buscam equidade, qualidade de vida e universalidade no acesso à saúde.

A centralidade e a importância da pesquisa epidemiológica têm sido reforçadas no século XXI. A produção científica nacional cresceu em volume e diversidade nas últimas décadas, consolidando grandes inquéritos de base populacional e o amplo uso de sistemas de registros oficiais. Mais recentemente, foi decisiva na produção de conhecimento sobre a COVID-19, quando mostrou capacidade de rápida mobilização, enfrentamento às campanhas de desinformação e apoio à sociedade na cobrança por políticas públicas efetivas. Esses avanços coexistem, contudo, com desafios que atravessam a ciência em escalas global e nacional: a difusão de discursos anticiência, a instabilidade dos recursos destinados à pesquisa^1^ e as desigualdades regionais na distribuição do financiamento e da capacidade instalada^2^. Também se impõe o debate sobre a contínua necessidade de nosso aperfeiçoamento enquanto pesquisadores — dos já consolidados às novas gerações — sobre avanços teórico-metodológicos nas pesquisas conduzidas e quanto aos limites do produtivismo acadêmico com sua distância em relação às necessidades sociais.

Ao mesmo tempo, abrem-se oportunidades para o fortalecimento do campo. O avanço das pesquisas alinhadas às necessidades da população, com formação crítica e qualificada dos pesquisadores, a adoção das práticas de ciência aberta^3^, a constituição de redes interinstitucionais no país e de parcerias internacionais, inclusive Sul–Sul, a aproximação dos pesquisadores das populações pesquisadas e o uso crítico e produtivo de inovações tecnológicas oferecem caminhos para expandir o alcance e a relevância social da pesquisa epidemiológica. A incorporação desses elementos, ancorada em referenciais teórico-metodológicos sólidos e guiada por princípios éticos e compromisso com a população, pode potencializar a capacidade da epidemiologia de produzir conhecimento transformador. A história da epidemiologia brasileira é muito rica e consistente, o que aumenta a responsabilidade de todos de continuarmos os avanços e construirmos um futuro no qual a ciência se mantenha crítica, socialmente referenciada e capaz de enfrentar as crises sanitárias e políticas que desafiam o país.

Refletir sobre os desafios e as oportunidades em pesquisa colocados aos epidemiologistas é central em nossa prática para permitir ações planejadas e com objetivo concreto e coletivamente construído. Por esse motivo, o V Plano Diretor para o Desenvolvimento da Epidemiologia no Brasil (2025–2029), construído com base em consultas à comunidade de epidemiologistas nas instituições acadêmicas, da gestão e dos serviços de saúde do Sistema Único de Saúde (SUS), em diversos debates, com dezenas de participantes e ao longo de dois anos, dedicou uma área temática à pesquisa em epidemiologia. Nela, três eixos centrais norteiam o diagnóstico dos desafios a serem enfrentados e as ações a serem desenvolvidas para superá-los:

- desigualdades sociais e iniquidades em saúde;
- financiamento para pesquisas e instituições de pesquisa;
- teoria, método e ética em pesquisa.

Neste artigo, que acompanha a publicação do V Plano Diretor para o Desenvolvimento da Epidemiologia no Brasil (2025–2029), alguns dos aspectos apresentados no documento são contextualizados e discutidos.

### Da instabilidade à consolidação de um projeto robusto de ciência no Brasil

A pesquisa científica é um dos pilares para o desenvolvimento econômico, social e tecnológico de qualquer nação. No Brasil, entretanto, o financiamento em ciência e tecnologia (C&T) enfrenta um cenário de instabilidade que tem sido marcado por avanços pontuais e frequentes retrocessos. Esses movimentos comprometem a continuidade de projetos, a consolidação de grupos de pesquisa e a manutenção de pesquisadores no país. A compreensão desse contexto exige um olhar histórico, político e institucional crítico, e a reversão dele demanda forte articulação de diferentes setores da sociedade, incluindo os epidemiologistas.

O Brasil construiu um arcabouço institucional importante na área de C&T ao longo de décadas. Entre os anos 2000 e 2013, houve um ciclo de expansão de financiamento com a criação dos fundos setoriais e a consolidação do Fundo Nacional de Desenvolvimento Científico e Tecnológico (FNDCT), ampliando os recursos disponíveis para o setor. Porém, em seguida, observou-se retração significativa nos investimentos. Entre 2013 e 2020, os recursos federais em C&T caíram 37% em termos reais^1^. Nesse último ano, os orçamentos do Conselho Nacional de Desenvolvimento Científico e Tecnológico (CNPq) e do FNDCT, somados, tornaram-se menores do que aqueles registrados no início dos anos 2000. Aliás, entre 2000 e 2020, o orçamento corrigido do Ministério da Ciência, Tecnologia e Inovação foi reduzido em 52% e do Ministério da Educação em 50%^1^. Já o dispêndio da Coordenação de Aperfeiçoamento de Pessoal de Nível Superior (Capes) retrocedeu a patamares próximos a 2011. Apesar de esforços de recomposição após esse período, como a aprovação da Lei Complementar nº 177/2021, que buscou assegurar a plena execução dos recursos do FNDCT, e melhorias em peças orçamentárias em 2023 e 2024 em comparação aos anos anteriores, ainda prevalecem desequilíbrios estruturais, instabilidade e subfinanciamento. A título de ilustração, entre 2021 e 2024 as despesas pagas pela Capes cresceram em termos absolutos apenas em R$ 1,2 bilhão^4^ e pelo CNPq em R$ 0,5 bilhão^5^.

Esse contexto nacional de retrocessos com avanços paliativos ocasiona o enfraquecimento da formação de pesquisadores e da base científica nacional, ao mesmo tempo que aprofunda as desigualdades na produção científica e capacidade de inovação entre as diferentes regiões do país, amplia a dependência externa e mina as condições do Brasil de transformar ciência em desenvolvimento soberano. Exemplos são a fuga de cérebros e a ausência de políticas estruturantes para fixação de jovens doutores. Mais do que recompor valores orçamentários ou dispor de programas pontuais, é necessário repensar o lugar da ciência na agenda pública, rompendo com a lógica de financiamento como variável de ajuste fiscal e afirmando-a como investimento estratégico.

Diante desse cenário, o V Plano Diretor para o Desenvolvimento da Epidemiologia no Brasil (2025–2029) define como uma das suas prioridades a luta pela criação de políticas mais robustas e estáveis de financiamento para pesquisas epidemiológicas e que se ampliem as pesquisas que investigam os impactos do investimento público em C&T no desenvolvimento do país. O que se necessita não são apenas acréscimos marginais na dotação orçamentária de agências e ministérios ou de retóricas de valorização da pesquisa científica. É indispensável um conjunto articulado de ações, tanto imediatas quanto de longo prazo, capazes de blindar a ciência das instabilidades políticas e de consolidá-la como eixo estruturante do desenvolvimento nacional, assegurando-lhe prioridade real na agenda pública.

Além de viabilizar financiamento robusto, garantir o acesso equitativo a ele é fundamental para promover diversidade, inovação e excelência científica. Apesar de avanços, persistem desigualdades tanto com relação a temas quanto a beneficiários. Essas disparidades prejudicam avanços em alguns temas negligenciados e também carreiras individuais, limitando a diversidade de perspectivas essenciais às descobertas científicas^6^.

Um exemplo claro se dá nos estudos das doenças tropicais negligenciadas no Brasil. Ao mesmo tempo que o financiamento da pesquisa para doenças tropicais negligenciadas estagnou ao longo dos anos no país, com financiamento insuficiente para doenças com alta prevalência, carga e taxa de mortalidade^7^, observa-se falta de correlação entre carga da doença, produção científica e financiamento governamental^8^. Por exemplo, entre os estudos publicados entre 2012 e 2016 sobre o tema, há ênfase na pesquisa biomédica básica e escassez de estudos sobre políticas, sistemas de saúde e ciências sociais.

Entre 2007 e 2016, os financiamentos governamentais para pesquisas sobre câncer no Brasil, que somaram quase US$ 489 milhões destinados a 7.622 projetos e 3.068 pesquisadores, apresentaram forte concentração regional: o sudeste recebeu 84,4% desses recursos^9^. Mais recentemente, dados do FNCDT mostram que 93% de seus recursos reembolsáveis estimados para 2023 de projetos contratados anteriormente se concentraram nas regiões Sudeste e Sul^10^. Entre os recursos não reembolsáveis de 2022 e 2023, verificou-se que de 69 a 75% dos valores contratados de projetos encerrados no período se concentraram no sul e sudeste, contra apenas 2 a 6% no norte^11^. Sua distribuição de bolsas em 2023 concentrava no sul e no sudeste 66% das bolsas de pós-doutorado júnior, 72% de iniciação científica e quase 60% de desenvolvimento tecnológico industrial^11^.

Em síntese, o chamado do V Plano Diretor para o Desenvolvimento da Epidemiologia no Brasil (2025–2029) é para que o Brasil assuma posição mais arrojada no financiamento em C&T, com um projeto estruturante e ambicioso. Propõe-se que consigamos deixar de legado para os anos seguintes uma proposta transformadora e sólida de C&T com amplo apoio social e menos vulnerável a ciclos de austeridade e de tentativas de desmonte.

### Agendas, métodos e sentidos da pesquisa em Epidemiologia no Brasil

Além do financiamento, precisamos debater amplamente em nossa área os temas que temos investigado e como temos desenvolvido esses estudos. Podemos considerar os congressos científicos como introdução, pois desempenham papel relevante na definição e na visibilidade das agendas de pesquisa. No caso da epidemiologia no Brasil, os congressos da Associação Brasileira de Saúde Coletiva (Abrasco) na área organizam a apresentação de trabalhos em eixos temáticos, que funcionam como um espelho das prioridades e dos desafios da área. A comparação entre os congressos brasileiros de epidemiologia de 2017 e 2024 mostra grande estabilidade desses eixos, como teoria e métodos em epidemiologia; avaliação de políticas e serviços; ensino e formação; saúde ao longo do curso de vida; doenças crônicas; violências e acidentes; e estudos em populações específicas (indígenas, negras, LGBTQIAPN+, privadas de liberdade, migrantes e refugiados). No período mais recente houve a inclusão ou a expansão de temas, como crise climática e saúde, emergências em saúde pública, envelhecimento e agravos relacionados ao trabalho. Apesar desses avanços, persistem lacunas importantes diante das necessidades emergentes e latentes da sociedade.

A consolidação de grandes pesquisas nacionais, como a Pesquisa Nacional de Saúde^12^, a Pesquisa Nacional de Saúde do Escolar^13^, a Pesquisa de Orçamentos Familiares^14^ e o Inquérito do Sistema de Vigilância de Fatores de Risco e Proteção para Doenças Crônicas por Inquérito Telefônico^15^ alavancaram, com grande relevância e necessidade, as pesquisas sobre as doenças crônicas não transmissíveis (DCNT) e seus cuidados e fatores de risco, sobretudo considerando os dois Planos de Enfrentamento às DCNT, do Ministério da Saúde, um com metas de 2011 a 2022^16^ e outro de 2021 a 2030^17^.

Além disso, temos alguns estudos longitudinais em andamento que constituem referenciais para a compreensão dos determinantes de saúde e doença. Como exemplos de vários outros estudos de extrema relevância, há a Coorte de Nascimentos de Pelotas, iniciada em 1982 e com novas ondas periódicas desde então, que acompanha indivíduos desde o nascimento possibilitando análises sobre desenvolvimento infantil, adolescência e vida adulta^18^; o Estudo Longitudinal de Saúde do Adulto (ELSA-Brasil), iniciado em 2008, voltado para a população adulta, com ênfase em DCNT, particularmente cardiovasculares e metabólicas^19^; e o Estudo Longitudinal da Saúde dos Idosos Brasileiros (ELSI-Brasil), que investiga condições de saúde, funcionais e sociais da população idosa^20^. Explorando bancos de dados oficiais do Brasil, há ainda o Centro de Integração de Dados e Conhecimentos para Saúde^21^.

Em contrapartida, agravos relacionados à saúde mental e às violências — temas considerados prioritários nas discussões do V Plano Diretor para o Desenvolvimento da Epidemiologia no Brasil (2025–2029) por sua alta e crescente carga na população brasileira e pelas centenas de milhares de afastamentos do trabalho — ainda dispõem de menos estudos diante de sua relevância epidemiológica. Apenas como exemplos, em 2015 esses transtornos foram responsáveis por 9,5% do total de anos de vida perdidos por morte ou incapacidade (DALY), ocupando a terceira e a primeira posição na classificação de DALY e anos de vida perdidos por morte prematura (YLD), respectivamente, com destaque para os transtornos depressivos e de ansiedade^22^. Segundo relatório do Ministério Público do Trabalho e do Escritório da Organização Internacional do Trabalho para o Brasil, os benefícios por incapacidade temporária associados à saúde mental no trabalho mais do que dobraram entre 2022 e 2024, passando de quase 201 mil para mais de 471 mil (aumento de 134%)^23^.

As estimativas das violências também são assustadoras. Segundo os dados do Sistema de Informação de Agravos de Notificação, apenas em 2023 foram registrados 588.388 casos de violência interpessoal/autoprovocada no Brasil^24^. Já segundo o Mapa de Segurança Pública de 2025, apesar de o Brasil ter registrado em 2024 redução de 6,3% nos homicídios dolosos em relação ao ano anterior (35.365 vítimas, quando foram 37.754 em 2023), a magnitude continua inaceitável, e a violência contra a mulher é crescente e manifesta-se em diferentes formas. O ano de 2024, por exemplo, registrou 2.422 homicídios de mulheres, 1.459 feminicídios e 71.834 casos de estupro de mulheres, o que corresponderia a uma média de 196 casos por dia.

Há muito tempo a permanência de alta carga de doenças infecciosas no Brasil e, mais recentemente, a pandemia de COVID-19 também reforçaram a importância dos estudos de vigilância e sobre doenças transmissíveis^25^, que merecem monitoramento constante e necessitam dele. Ao mesmo tempo que as doenças negligenciadas continuam desafiadoras^26^, temas de investigação como mudanças climáticas^27^ e emergências em saúde pública^28^ também precisam ter suas pesquisas ampliadas. É importante desenvolver estudos em temas como resposta a desastres — surtos e epidemias, em diálogo com vigilância e incluindo a sociedade —, descrição e avaliação de políticas da saúde de pessoas privadas de liberdade, saúde do trabalhador formal e informal, saúde indígena, quilombola e de populações tradicionais, doenças transmissíveis emergentes e reemergentes, saúde mental, e violência e outras causas externas. É desafiador, mas necessário, viabilizar essa ampliação, ao mesmo tempo que fortalecemos as pesquisas de doenças crônicas, que correspondem à maior proporção de óbitos no país.

Outro aspecto que merece atenção é a contínua necessidade de avanços em modelos teóricos capazes de sustentar e orientar a interpretação dos dados epidemiológicos. É essencial que pesquisas não se apoiem em aplicações mecânicas de modelos estatísticos e com pouca reflexão conceitual, algo que impede a contextualização dos achados e a ampliação de sua relevância social^29-32^. Na área de desigualdades sociais em saúde, particularmente, é importante destacar que a produção científica não pode se limitar ao mero teste de significância estatística nas associações entre variáveis socioeconômicas e desfechos em saúde. Estudo anterior mostrou que, apesar de sua presença crescente na literatura, a área de desigualdades em saúde ainda apresenta escassez de abordagens longitudinais, multidimensionais e interdisciplinares, dificultando uma compreensão mais profunda dos mecanismos que produzem e reproduzem iniquidades^30^.

Nessa direção, os testes estatísticos, parte importante das análises epidemiológicas, precisam estar subordinados a um modelo teórico, a uma compreensão das questões econômicas, sociais e culturais que moldam as relações estudadas. O entendimento das causas e dos obstáculos que sustentam as desigualdades sociais em saúde exige mais do que a importante descrição empírica das disparidades; é preciso procurar compreender suas forças motoras e consequências. A teoria, nesse contexto, não é acessória, mas essencial para orientar análises e subsidiar ações voltadas à equidade^33^. De modo especial, é preciso compreender criticamente os efeitos na saúde populacional dos processos de concentração de renda e da implementação de políticas neoliberais e de austeridade, além daquelas iníquas e ineficientes. Esse esforço envolve a formação sólida em teoria e análise de dados nos programas de pós-graduação — com pesquisadores críticos que trabalhem de forma transdisciplinar e criativa —, a consolidação de agendas de investigação que proponham avanços conceituais e metodológicos e o fortalecimento de pesquisas com análises críticas, inovadoras, robustas e teoricamente fundamentadas.

Já as pesquisas sobre desigualdades de raça em saúde ou racismo e saúde se encontram ainda em estado de desenvolvimento, com aumento na última década^30,32^. Segundo estudo^32^, as áreas com mais publicações sobre a saúde da população negra foram a saúde pública, com 21,6% do total de estudos analisados, enfermagem, saúde coletiva, ciências sociais e ciências sociais aplicadas e psicologia. A epidemiologia contou com 6% do total de pesquisas no período analisado. Os estudos que abordam as desigualdades sociais em saúde muitas vezes inserem a raça/cor da pele em suas análises, mas são menos comuns os que têm como foco central o efeito da raça/cor da pele na saúde. Portanto, vale dizer que, embora as pesquisas tenham avançado substancialmente nesse tema, ainda são necessários investimentos em estudos na área e uma visão crítica do racismo na sociedade.

Vale também relatar a escassez de investigações com grupos específicos ou em estado de vulnerabilização, como a população indígena^34^, quilombola, ribeirinha^35^ e LGBTQIAPN+. Ainda que dois suplementos temáticos de 2024, um da *Revista Brasileira de Epidemiologia* ^36^ e um da revista *Epidemiologia e Serviços de Saúde* ^37^, tenham sido exclusivamente destinados à saúde da população trans, é importante expandir a produção científica com grupos vulnerabilizados. Em geral, é nosso compromisso pensar nas pesquisas não sobre alguns grupos populacionais, mas sim com e para eles.

Enquanto um aspecto desses desafios envolve o financiamento em pesquisa e a construção coletiva da agenda nacional de prioridades, outra parte se refere ao incentivo a um produtivismo acadêmico que muitas vezes se apresenta desconectado das necessidades da sociedade. Há muito tempo se acumulam críticas quanto às limitações de indicadores bibliométricos que avaliam o "impacto" da produção científica apenas por citações^38,39^. Também são contestados os efeitos deletérios que critérios de avaliação essencialmente baseados no número de publicações podem ter sobre a autonomia, a qualidade, a agenda de pesquisa dos pesquisadores e a sua saúde^40^. Um aspecto preocupante igualmente há algum tempo é a proliferação de pesquisas que não buscam a originalidade^41^, a diversificação ou a criatividade, pois o ambiente acadêmico e suas recompensas estão mais fortemente amarrados à quantidade publicada e menos à qualidade, pertinência ou inovação. Embora possamos ser prolíficos e dinâmicos na produção científica, afinal é nossa missão produzir e difundir conhecimento, nossas pesquisas devem ser guiadas pelas necessidades da população e pelo compromisso de contribuir efetivamente com o avanço do conhecimento. Para isso, precisamos de ambientes colaborativos, redes de pesquisa interinstitucionais e financiamento robusto. Nesse sentido, precisamos unir nossos esforços para construir políticas de avaliação e de reconhecimento científico baseados nos desafios concretos de nossa sociedade. Isso envolve repensar a avaliação de programas de pós-graduação, os processos seletivos para docentes e pesquisadores, as linhas de fomento e critérios de seleção de projetos, assim como as revistas nas quais publicamos nossos achados. Ao menos desde o quadriênio 2017–2020, na área de saúde coletiva os critérios de avaliação da Capes vêm priorizando a qualidade da formação e o impacto social dos programas de pós-graduação. Mais recentemente, passou-se a incluir também a avaliação qualitativa das publicações científicas do corpo docente. Tais mudanças têm o potencial de induzir melhorias na produção intelectual e na formação dos futuros pesquisadores. Precisamos continuar avançando e construindo coletivamente os próximos passos de modo a buscar o alinhamento de nossos objetivos com nossa práxis.

### Conhecimento em movimento: ciência aberta, tradução e democratização na epidemiologia brasileira

O Brasil ocupa posição de destaque na produção científica em saúde e epidemiologia, conforme já comentado, no entanto uma contradição persiste: parte desse conhecimento não se dissemina fora do meio acadêmico e em muitos casos não se traduz em decisões políticas ágeis, efetivas e equânimes. Como dito anteriormente, parte do desafio está na construção de uma agenda de acordo com as necessidades sociais. Todavia, outra face envolve a necessidade de se apresentar aos gestores, profissionais de saúde, movimentos sociais e à sociedade em geral a produção da ciência como algo compreensível e aplicável. Embora epidemiologistas brasileiros produzam pesquisas de alta qualidade, socialmente comprometidas e em volume admirável — mesmo diante de enormes desafios estruturais —, é essencial criar mecanismos eficazes de tradução e disseminação do conhecimento capazes de fazê-lo chegar até as pessoas e ter significado para diferentes atores. Democratizar a epidemiologia, portanto, não se restringe a abrir dados ou publicar artigos, mas exige repensar a velocidade de produção científica, formatos de comunicação, criar repositórios confiáveis e acessíveis e investir em métodos de popularização da ciência.

Em alguns casos, o tempo da ciência não acompanha a urgência da formulação de políticas públicas, o que pode contribuir para decisões baseadas em impressões ou em informações incorretas, e não em evidências. Embora muitas pesquisas demandem um longo período de desenvolvimento, é essencial que a comunidade científica seja capaz de oferecer respostas rápidas que atendam a necessidades prementes, seja em situações de emergências em saúde pública, tais como surtos, pandemias, desastres naturais, seja em qualquer outra necessidade de interesse social.

Superar essas barreiras exige compreender a tradução do conhecimento como prática estruturante da epidemiologia, o que significa desenvolver linguagens, produtos e métodos capazes de tornar as evidências epidemiológicas acionáveis por diferentes públicos. Para além dos artigos científicos, destacam-se relatórios executivos e painéis interativos para gestores, *podcasts*, vídeos e infográficos para a sociedade, materiais adaptados para escolas e coletivos comunitários aproximando a pesquisa de seus destinatários, reconhecendo-os como coprodutores de sentido e de soluções. O V Plano Diretor para o Desenvolvimento da Epidemiologia no Brasil (2025–2029) de Epidemiologia, nesse sentido, enfatiza a necessidade de se formar profissionais em técnicas de popularização científica, ampliando a visibilidade, a transparência e a circulação do conhecimento para além dos muros acadêmicos.

Ao mesmo tempo, devemos pensar nas formas de disseminação dos artigos científicos. Será importante ampliarmos as discussões e as ações em direção à ciência aberta, envolvendo aspectos políticos e técnicos relativos ao uso de repositórios confiáveis de dados epidemiológicos, protegidos pela Lei Geral de Proteção de Dados Pessoais (LGPD). Práticas para aumentar a transparência e a reprodutibilidade da ciência a fortalecem e a legitimam ainda mais. Por isso, precisamos ter em nosso horizonte a ampliação da publicação em acesso aberto, o compartilhamento de rotinas de análise de dados e de bancos de dados e a transparência em processos de revisão. O Brasil, especialmente pela iniciativa Biblioteca Eletrônica Científica Online (SciELO), tem forte histórico em publicações com acesso aberto. As revistas de saúde coletiva editadas no Brasil e que publicam a produção da epidemiologia brasileira carregam em sua missão a ampla difusão do conhecimento, no entanto acesso aberto nem sempre significa equidade ou justiça na disseminação científica. Expressivo volume de recursos públicos brasileiros tem sido direcionado ao pagamento de taxas de publicação em revistas estrangeiras, reorientando investimentos na ciência brasileira a grandes conglomerados empresariais. Ao mesmo tempo, editais de apoio à editoração do CNPq disponibilizam à média anual a cada revista brasileira contemplada o valor equivalente ao custo de duas taxas de processamento de artigos em revistas estrangeiras^42^. Repensar a disseminação do conhecimento produzido no Brasil, conciliando a importante internacionalização de nossa produção, sua tradução e aplicação na nossa sociedade, o fortalecimento das revistas editadas no país e o uso justo e racional do recurso público e a aproximação da sociedade, é missão da ciência brasileira, na qual a epidemiologia deve ter papel central.

Se o acesso amplo às publicações constitui um passo fundamental para democratizar o conhecimento científico, igualmente essencial é garantir o acesso aberto, democrático e transparente a dados secundários de interesse epidemiológico em toda a sua abrangência. Essa prática fortalece o controle social, amplia a legitimidade das políticas e cria condições para uma rica pluralidade de análises por diferentes atores sociais. Especialmente em pesquisas financiadas com recursos públicos e nas bases de dados de registros oficiais, é preciso que os órgãos públicos empreendam esforços para viabilizar acesso amplo aos dados — sejam de um sistema de informação, sejam de uma pesquisa específica, sejam derivados da vinculação (*linkage*) de bases de dados. É importante que a forma de acesso a eles seja aberta a todos os pesquisadores e grupos de pesquisa com interesse legítimo. Para isso, o debate com a comunidade científica e o controle social devem ser contínuos, e a segurança dos dados, conforme prevista na LGPD, deve ser garantida. De igual importância, o V Plano Diretor para o Desenvolvimento da Epidemiologia no Brasil (2025–2029) reforça que projetos de pesquisa financiados pelo Ministério da Saúde e outros órgãos públicos sejam prioritariamente selecionados por meio de editais públicos amplamente divulgados, enquanto as contratações diretas de grupos de pesquisa sigam critérios claros e públicos restritos a situações específicas e estratégicas.

Há, no país, oportunidade singular com a consolidação da Rede Nacional de Dados em Saúde, com iniciativas importantes na saúde digital, amplo uso de registros eletrônicos em diferentes níveis de atenção à saúde, extensas pesquisas domiciliares e nos serviços de saúde, somados ao desenvolvimento de técnicas analíticas para grandes bancos de dados, inteligência artificial e aprendizado de máquina. Associar essas potencialidades com o aprofundamento da democratização no acesso seguro e responsável aos dados e a perguntas de pesquisa relevantes para a sociedade brasileira é um saudável desafio sobre o qual a epidemiologia deve se debruçar.

De igual forma, é necessário enfrentarmos a assimetria de possibilidades entre regiões e municípios na análise dos dados. Enquanto alguns contam com equipes formadas para interpretar dados complexos, outros enfrentam dificuldades técnicas para o uso estratégico das informações disponíveis. Nesse vácuo, políticas mal orientadas e a desinformação encontram terreno fértil, reduzindo o potencial de impacto da produção epidemiológica. Por isso, é importante apoiar a educação permanente das equipes de vigilância e demais profissionais de saúde para coleta e entrada de dados nos sistemas de informação, incentivar a consolidação dos centros de informações estratégicas em vigilância e estimular a integração entre municípios, regionais de saúde e estados. Paralelamente, apoiar jovens pesquisadores e profissionais com inserção nos serviços públicos de saúde a elaborar e submeter propostas competitivas em editais de fomento.

Democratizar o conhecimento epidemiológico é um compromisso político com a sociedade brasileira, com as políticas sociais do país e com a redução das desigualdades. Significa garantir que a ciência oriente políticas de forma mais ágil e efetiva, mas também que seja apropriada por aqueles que vivem em territórios vulnerabilizados e por coletivos historicamente invisibilizados. A epidemiologia brasileira tem condições de liderar essa transformação, sendo importante que incorpore a tradução do conhecimento e a ciência aberta como eixos de sua prática.

## CONCLUSÃO

Diante dessas reflexões, a pesquisa em epidemiologia deve ser compreendida como expressão de um compromisso social que passa pelo domínio científico e vai além. Ela deve ser um caminho para se propor uma integração entre academia, serviços de saúde, gestores e sociedade, para que se possa consolidar uma ciência pública, crítica e transformadora.

A pesquisa em epidemiologia no Brasil se encontra diante do desafio de superar as limitações estruturais impostas pelo subfinanciamento e pelas desigualdades históricas, ao mesmo tempo que amplia sua capacidade de produzir conhecimento crítico, com qualidade, e inovador, socialmente referenciado e orientado para a transformação. O V Plano Diretor para o Desenvolvimento da Epidemiologia no Brasil (2025–2029) aponta alguns caminhos que procuram o fortalecimento da epidemiologia e convida toda a comunidade científica e a sociedade, ultrapassando fronteiras institucionais e disciplinares, a construir de forma colaborativa e solidária uma ciência que una inovação, rigor técnico e compromisso social.

## Data Availability

não se aplica.
