# Supplementary material for: ERRATUM: Research in Epidemiology in Brazil: challenges, pathways, and commitments for the future
Source: Rev Bras Epidemiol. 2026 Mar 9;28(Suppl 1):e250003supl1erratum. doi: 10.1590/1980-549720250003.supl.1erratum (PMC12971064; doi:10.1590/1980-549720250003.supl.1erratum)
Supplement: Related texts

# Correction: ERRATA: Pesquisa em epidemiologia no Brasil: desafios, caminhos e compromissos para o futuro.

**DOI:** 10.1590/1980-549720250003.supl.1.2erratum

No artigo "Pesquisa em epidemiologia no Brasil: desafios, caminhos e compromissos para o futuro", DOI: https://doi.org/10.1590/1980-549720250003.supl.1.2, publicado no periódico Rev. bras. Epidemiol. 28 (suppl 1) • 2025.

**Onde se lia:**

Antonio Fernando Boing, Maria de Jesus Mendes da Fonseca, Margareth Guimarães Lima, Claudia Leite de Moraes, Katia Vergetti Bloch

**Leia-se:**

Antonio Fernando Boing, Maria de Jesus Mendes da Fonseca, Margareth Guimarães Lima, Katia Vergetti Bloch, Claudia Leite de Moraes

Editora Científica: Maria Fernanda Tourinho Pereshttps://orcid.org/0000-0002-7049-905X
